# Comparison between ultrasound-guided TIVAD via the right innominate vein and the right internal jugular vein approach

**DOI:** 10.1186/s12893-019-0651-0

**Published:** 2019-12-11

**Authors:** Xingwei Sun, Xuming Bai, Jiaofeng Shen, Ziyang Yu, Zhixiang Zhuang, Yong Jin

**Affiliations:** 1 Department of Intervention, The Second Affiliated Hospital of Soochow University, Suzhou, 215004 Jiangsu China; 2 Department of Oncology, The Second Affiliated Hospital of Soochow University, Suzhou, 215004 Jiangsu China; 3 Department of Ultrasound, The Second Affiliated Hospital of Soochow University, Suzhou, 215004 Jiangsu China

**Keywords:** Totally implantable venous access device, Innominate vein, Internal jugular vein, US-guided, Cancer

## Abstract

**Background:**

To compare the efficacy and safety of right internal jugular vein (IJV) approach and right innominate vein (INV) approach for US-guided totally implantable venous access devices (TIVADs), and to explore the advantages and disadvantages of the two approaches.

**Methods:**

Six hundred and nineteen adult patients had long-term infusion and chemotherapy needs and inconvenience of peripheral venous infusion. Right INV approach was used to implant 339 cases of TIVADs, and right IJV approach was used to implant 280 cases of TIVADs. The success rate of one-time catheterization and the incidence of complications in the two groups were retrospectively analyzed.

**Results:**

All patients were successfully implanted in TIVAD. The success rates of one-time puncture in INV group and IJV approach group were 98.53% (334/339) and 95.36% (267/280), respectively. There was significant difference between the two groups (*P* = 0.020). The incidence of perioperative complications and long-term complications in the right INV group were 1.18% (4/339) and 3.54% (12/339), respectively, while those in the right IJV group were 1.43% (4280) and 3.93% (11280). There was no significant difference in the incidence of perioperative or long-term complications between the two groups (*P* = 0.785, *P* = 0.799, respectively).

**Conclusions:**

US-guided TIVADs via the right INV approach and the right IJV approach are both safe and reliable. The right INV approach improves the one-time puncture success rate, as long as the technique is properly operated, serious complications rarely occur.

## Background

The application of a totally implantable venous access device (TIVAD) is regarded as a leap-forward development of infusion technology, and it significantly reduces the risk of drug infusions, especially chemotherapeutic drug infusions [1]. TIVAD has been widely used in clinical application, which is superior to other long-term intravenous infusion pathways and is the best choice for cancer patients [2].

In 1982, Niederhuber et al. [3] first placed TIVADs via the cephalic vein through surgical techniques. Nowadays, internal jugular vein (IJV) approach has become the most commonly used approach in clinical application because of its high success rate and low complications [4, 5].

In recently, with the continuous development of ultrasound (US) technology, the INV approach is gradually being considered and applied in clinical practice [6–8]. However, the reports of US-guided INV approach for TIVADs implantation in adult patients are limited [9].

In the previous research, we introduced the safety and feasibility of US-guided TIVADs via the right INV approach [10, 11]. In this study, a total of 619 cases managed by US-guided TIVADs through the right INV or right IJV approach in our department were retrospectively compared and analyzed consecutively.

## Methods

This study was approved by the Ethics Committee, and written informed consent was obtained from all the cases. The study cohort included adult patients receiving US-guided TIVADs through the right INV or right IJV approach from January 2016 to January 2018.

For the purposes of the present study, the success rate of operation, time of operation, success rate for the one-time puncture, catheterization length, and intraoperative and postoperative complications were recorded.

Clinical data were acquired from the operation and nursing records.

### Materials

BardPort (8,806,061, 6F; UT, USA) and a LOGIQe ultrasound device (General Electric, Fairfield, CT) was used in all cases.

## Methods

Sterile procedures were strictly followed during the surgery. The operation was performed by two experienced interventional physicians for our team.

**Fig. 1:**
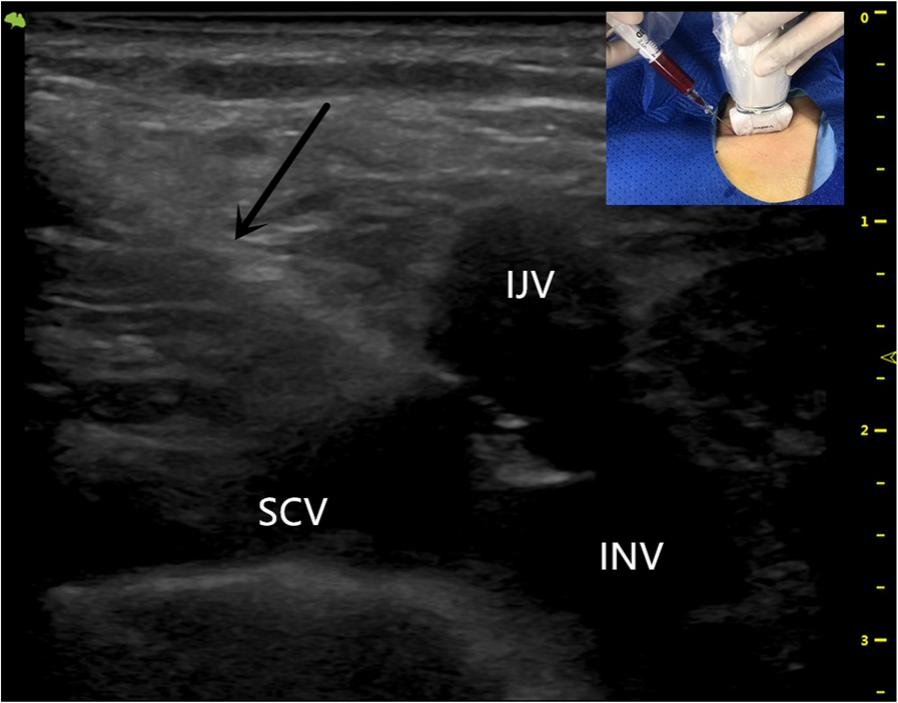
Ultrasound-guided successful puncture of right INV with inserting needle (black arrow). INV longitudinal view, in-plane approach. INV indicates innominate vein; IJV indicates internal jugular vein; SCV indicates the subclavian vein

**Fig. 2:**
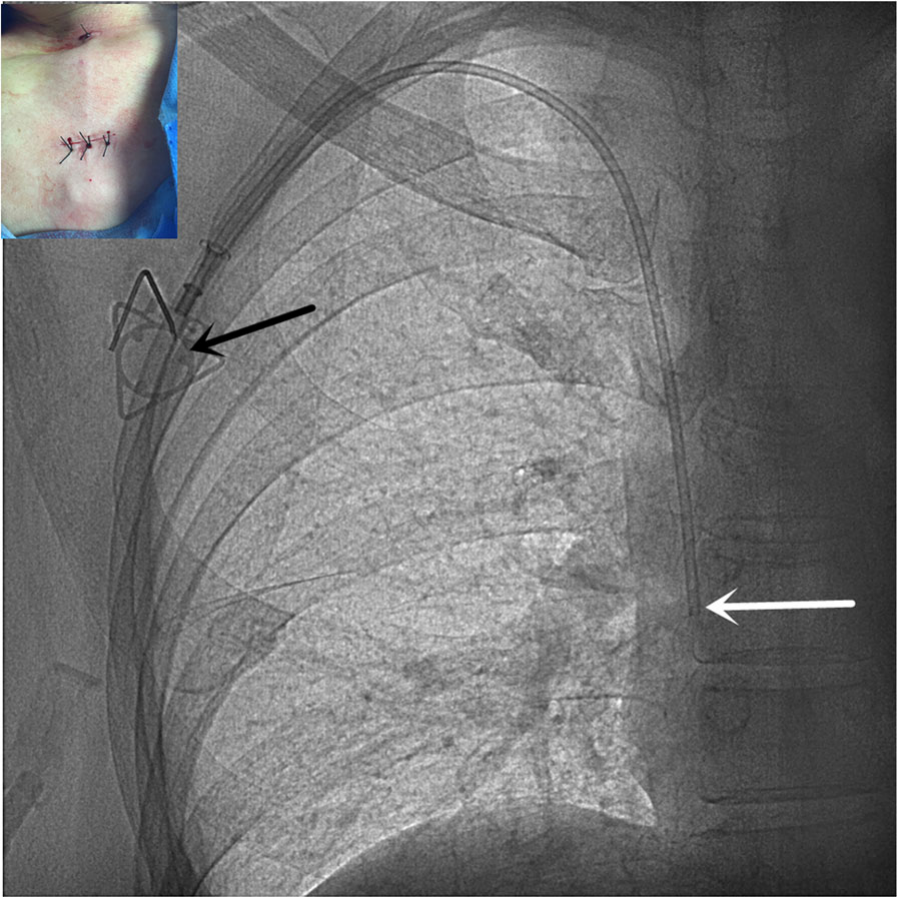
The catheter crosses over the clavicle and enters the superior vena cava via the right INV. The port (black arrow) is located on the right chest wall, and the tip of the catheter (white arrow) is located at the junction of the superior vena cava and the right atrium

**Fig. 3:**
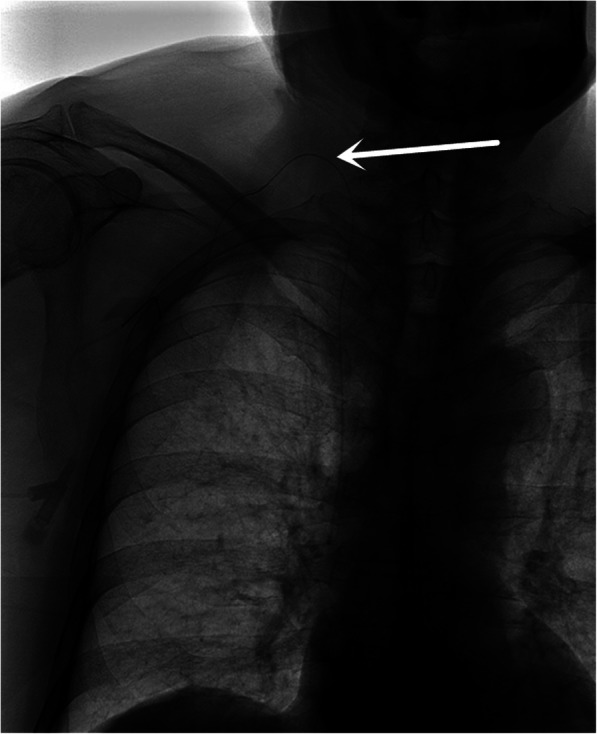
TIVAD is implanted via the right IJV. The catheter crosses the clavicle and reversed into the right IJV. The white arrow indicates the angle at which the catheter folds

The right INV TIVADs was performed as follows:

1. The patient is in a supine position with the head turned to the left。
2. The scope of disinfection should be more than 15 cm above the intended pipe and TIVAD site, and sterile sheet should be laid.
3. The high-frequency ultrasound probe runs down the right IJV to the sternoclavicular joint to get good view of the right INV. Local anesthesia was performed at the puncture site with 1% lidocaine. After successful puncture with in-plan technique (Fig. 1), it enters the guide wire, sheath, and catheter sequentially.
4. Skin incision was made at the ipsilateral subclavian 2-3 cm, subcutaneous tissue was bluntly separated, and appropriate size pocket was made.
5. Using the subcutaneous tunnel needle, the catheter was subcutaneously introduced into the pocket from the puncture site. The tip of catheter was located at the junction of superior vena cava and right atrium under fluoroscopy. (Figure. 2).
6. Cut off the catheter and connect the catheter to the port. After local hemostasis, incisions were sutured and X-ray images of TIVAD were retained (Fig. 3).

### Maintenance of TIVADs

The maintenance and management of the infusion port were performed by specialized nurses, and 10-ml flushing tube of 50-100iu /ml heparin saline was used, not more than once in every 28 days.

### Data collection

Success rate of the one-time puncture: the one-time is considered to be successful if the intraoperative puncture needle enters the INV or IJV and successfully enters the guidewire without a secondary puncture of skin.

Perioperative complications: arterial puncture, pneumothorax, hemothorax, local hematoma formation, incision infection.

Postoperative complications: complications occurred two weeks after implantation, including thrombosis, fibrinolysis, pinch-off Syndrome, catheter malposition, catheter rupture, catheter-associated infection, port inversion, local skin rupture.

### Statistical analysis

SPSS19.0 statistical software was used to statistically analyze the surgical details and postoperative complications of the two groups. The measurement data were represented by Mean ± SD, and T test was used for comparison between the two groups. The ratio of counting data indicated that chi-square test was used for the comparison between the two groups. *P* < 0.05 was considered statistically significant.

## Results

The general data between the two groups are summarized in Table 1. There was no statistical difference between the two groups in gender, age, height and weight.

**Table 1:** patient’s characteristics

	Overall (*N* = 619)	INV(*N* = 339)	IJV(*N* = 280)	*P* value
Age (years)	52.57 ± 14.63	54.75 ± 14.62	51.20 ± 11.53	0.812
Male / Female	242/ 377	128 / 211	114/166	0.453
Height (cm)	165.43 ± 12.37	165.14 ± 10.49	167.10 ± 13.04	0.980
Weight (kg)	56.64 ± 14.12	55.03 ± 12.05	58.66 ± 9.19	0.905
Body Mass Index	19.64 ± 3.69	20.03 ± 4.11	19.20 ± 3.15	0.763
Prothrombin Time (seconds)	13.23 ± 3.52	12.23 ± 3.18	14.45 ± 2.29	0.041
Activated Partial Thromboplastin Time (seconds)	42.18 ± 10.01	41.20 ± 7.12	43.34 ± 9.01	0.670
Breast cancer (%)	256 (41.36)	141 (35.34)	115 (35.71)	0.896
Liver cancer (%)	154 (24.88)	86 (25.39)	68 (24.29)	0.756
Lung cancer (%)	128 (20.68)	75 (22.12)	53 (18.93)	0.329
Gastric cancer (%)	67 (10.82)	30 (8.85)	37 (13.21)	0.082
Rectal cancer (%)	15 (2.42)	7 (2.06)	8 (2.86)	0.523

Details of TIVADs procedure are summarized in Table 2. All patients underwent successful surgery. The success rate of one-time puncture in the right INV group was 334 (98.53%) and that in the right IJV group was 267 (95.36%). There was significant difference between the two groups (*P* = 0.020).

**Table 2:** Showing details of TIVADs procedure and insertion duration

	Overall (*N* = 619)	INV(*N* = 339)	IJV(*N* = 280)	*P* value
Success rate of one-time attempt (N)	97.09% (601)	98.53% (334)	95.36% (267)	0.020
Operation time (minutes)	29.24 ± 6.37	28.15 ± 5.97	30.92 ± 6.26	0.303
Length of catheter introduction (cm)	21.39 ± 6.17	19.64 ± 4.37	23.47 ± 6.31	<0.01
Port-carrying time (days)	295.23 ± 36.19	312.09 ± 48.10	278.51 ± 35.85	0.039

All 619 patients in this study were successfully implanted in TIVADs. The depth of catheterization (from the puncture point to the tip of the catheter) was ll-16 cm. The mean length of catheter insertion was 19.64 ± 4.37 cm (range of 16–21 cm) in the right INV group and 23.47 ± 6.31 cm (range of 19–26 cm) in the right IJV group, and the difference was statistically significant (*P*<0.01).

Perioperative complications were observed in 4 cases (2 artery puncture, 1 local hematoma and 1 pneumothorax) in the right INV group and 4 cases (3 artery puncture and 1 pneumothorax) in the right IJV group (Table 3). There were no hemopneumothorax and lymphatic duct injury in both groups. Perioperative complications rate did not have any significant difference (1.18% vs 1.43%, *P* = 0.785). None of these complications required any specific intervention except compression of the puncture site. The complete cannulation procedure was then repeated or changed the puncture site successfully.

**Table 3:** Incidence of intraoperative and postoperative complications and processing measures (*N* = 619)

Complications	INV(*N* = 339)	IJV(*N* = 280)	*P* value	Intervention
Artery puncture (%)	2 (0.59)	3 (1.07)		Self-limiting
Local hematoma (%)	1 (0.29)	0		Compression
Pneumothorax (%)	1 (0.29)	1 (0.36)		Self-limiting
All preoperative complications (%)	4 (1.18)	4 (1.43)	0.785	
Poor healing of the incision(%)	1 (0.29)	0		Secondary suture
Catheter-related infection (%)	4 (1.18)	2 (0.71)		Antibiotics, catheter removal
Thrombosis (%)	1 (0.29)	2 (0.71)		Anticoagulation, catheter removal
Fibrous sheath (%)	6 (1.77)	4 (1.43)		Thrombolysis Catheter removal
Catheter fracture (%)	0	1 (0.36)		Catheter removal
Catheter malposition (%)	0	2 (0.71)		Catheter removal
All Postoperative complications (%)	12 (3.54)	11 (3.93)	0.799	

The Port-carrying time in the right INV group and IJV group was (312.09 ± 48.10) days and (278.51 ± 35.85) days, respectively, with significant difference (*P* = 0.039).

Post-procedure complications happened in 12 patients in the INV group (poor healing of the incision in 1case, thrombosis in 1 case, catheter-related infection in 4 cases, fibrin sheath formation in 6 cases) and 11 patients in the IJV group (catheter-related infection in 2 cases, thrombosis in 2 cases, fibrin sheath formation in 4 cases, catheter malposition in 2 cases, and catheter fracture in 1 cases). Differences between groups about postoperative complications rate were not significant (3.54% vs 3.93%, *P* = 0.799) **(**Table 3**)**.

## Discussion

This is the first study on a large sample comparing in-plane US-guided supraclavicular right INV approach with right IJV approach for TIVADs in adults. Our main finding is that the INV technique had a significantly higher first-attempt success rate compared with IJV, and INV approach had lower perioperative and postoperative complications, although the differences between groups about complication rates were not significant. US-guided TIVADs via the right INV approach and the right IJV approach are both safe and reliable.

The methods of TIVADs implantation mainly include venous cutdown and venous puncture [12]. At present, percutaneous puncture for TIVADs implantation via IJV and SCV are the most widely used [4]. However, the IJV and SCV may not be the best option for many clinical situations.

TIVADs via IJV approach has a high puncture point and a large folding angle of the catheter **(**Fig. 3**)**. This may lead to reduction of comfort, unattractive appearance and even lead to discount and rupture of the catheter [13–15]. The SCV approach is more convenient and comfortable than IJV, but SCV approach has the possibility of occurrence of pinch-off syndrome (POS), which is the main cause of catheter rupture [16].

Cephalic vein cut-down technique has been used for more than 30 years [5]. According to the previous studies, in comparison with the SCV approach, the incidence of complications of cephalic vein approach using surgical techniques for TIVAD is lower, and it is considered superior to the SCV approach [17–19]. KOKETSU et al. [20, 21] also believe that TIVAD can provide safe and feasible infusion channels for patients through cephalic vein, which is worthy of promotion and application. However, surgical techniques for TIVAD also have the disadvantages of long operation time, low success rate, and great trauma.

The IJV merges with the SCV behind the sternoclavicular joint to form the INV, the bilateral INVs converge to form the superior vena cava (SVC). We know that the INV is relatively fixed and has a larger diameter than the IJV and SCV, this provides the possibility of US-guided puncture of INV safely and effectively.

With the development of ultrasound technology, ultrasound-guided INV catheterization has been gradually applied in central vena catheterization (CVC) for almost 10 years, and many studies have confirmed its safety and effectiveness [6, 7, 9]. Early studies about INV catheterization were mainly focused on catheterization for neonates, possibly because of the small diameter of IJV in newborns than the adults and is more difficult to puncture; therefore, the INV approach was adopted.

Studies have shown that the success rate of left INV catheterization in infants and newborns is higher than that in right [22, 23]. However, in adults, the left INV is deeper and more variable than the right, and it is difficult to identify the thoracic duct by ultrasound [11].

In this study, we chosed to puncture the right INV to avoid lymphatic leakage caused by thoracic duct injury, as reported in our previous study [10, 11]. .But in Beccaria’s study [9], 78 patients with left INV catheterization did not have thoracic duct injury. Another study showed that left INV catheterization was safe and feasible in children [24]. Further studies are required to confirm the safety and feasibility of left INV approach of TIVADs for adult patients.

In this study, the success rate of one-time puncture in the right INV group was slightly higher than that in the right IJV group (98.64% vs 95.34%, *P* = 0.020) (Table 2). This was related to many anatomical advantages of right INV, such as thin subcutaneous tissue, large diameter and clear ultrasound display.

The success rate of the first puncture is related to the personal experience, besides, the ectopy of guidewire during puncture is also an important factor for the reduction of the rate of the first puncture of the internal jugular vein. In the right IJV group, the first puncture failed in 9 cases, among which 4 cases were confirmed to enter the ipsilateral SCV by DSA, and the guide wire was successfully adjusted. In the right INV group, there was no guide wire ectopy during puncture due to anatomical factor: Y-shaped anatomical morphology of IJV, SCV, and INV [6].

The success rate of the first puncture of INV reported in this study was higher than that of Beccaria et al. [9] (90.18%, 257/285), which might be related to the left INV approach used in Beccaria’s study.

Perioperative complications were mainly related to mechanical injury of central vein puncture. In this study, intraoperative complications rate in the right INV group was lower than that of IJV group although the differences were not statistically significant (1.18% vs 1.43%, *P*>0.05) (Table 3).

Perioperative complications were observed in 4 cases in the right INV group and in 3 casesin the right IJV group. The miss punctured artery was identified as right subclavical artery (SCA) or right common carotid artery (CCA) by ultrasound, and two patients were diagnosed as the small amount of pneumothorax by fluoroscopy during the operation. These self-limiting complications were not treated except for puncture site compression. The complete cannulation procedure was then repeated or changed the puncture site successfully.

The port-carrying time was mainly affected by the subjective factors of the patients. By the end of this study, 209 patients had their TIVADs removed autonomously or unplanned, among which 84.67% (177/209) of the patients thought that there was no need to use the infusion port in about 1 year. In addition, 5% (14/280) of patients in the right IJV group were admitted for the removal of TIVADs due to local foreign body sensation or neck discomfort, while this condition was 2.06% (7/339) in the INV group.

The overall incidence of postoperative complications in the study was 3.72% (23/619) **(**Table 3**)**, which was consistent with that reported in most other studies [25, 26]. Postoperative complication rate of the right INV group was 3.54% (12/339), which was lower than that of the right IJV group (3.93%, 11/280), although the differences were not statistically significant (*P* = 0.799).

Postoperative complications mainly included poor healing of the incision, catheter-related infection, thrombosis, fibrin sheath formation, catheter malposition, and catheter fracture, and most of these lead to an unplanned remove after the failure of processing measures **(**Table 3**)**.

It is worth noting that there is no catheter malposition or catheter rupture in the right INV group, and the mechanism for the low incidence of catheter malposition or catheter rupture are still unclear.

Compared with JIV approach, the INV approach has the advantages of low mobility, smooth catheter shape, and it avoid POS with supraclavicular approach, these are important factors that reduce catheter malposition or fracture of the catheter [27–29]. In addition, Y-shaped anatomical morphology of IJV, SCV, and INV is also a very important factor of INV approach to reduce catheter malposition.

The findings of our study have led to changes in our daily clinical practice. As a result of the high success rate of puncture, low rate of complications with right INV, we now prefer the right INV to IJV for TIVADs.

Considering its anatomical characteristics, we avoid the right INV in some conditions. Considering the need for surgery and radiotherapy, we do not allow patients with right breast cancer. Patients with not controlled local infection in the port area or the acute infection is not controlled effectively was not allowed. The catheter crosses the clavicle, and the emaciated patients are more likely to expose the catheter. The presence of coagulopathy should be considered as a relative contraindication.

Although this is a large sample comparison study with 619 enrolled patients, the study has certain limitations. First, this study was a retrospective review and some data were not accessible. Second, our incidence of postoperative complications might not be accurate, as we might have missed some TIVADs inserted at our hospital but removed in other institutions. Third, it was a monocentric study and all the TIVADs enrolled were of right INV approach or right IJV approach, the data of TIVADs with left INV approach are lacking, which may provide the further reference for clinicians. According to 2014 statistics, there were more than 400,000 cases of infusion port implantation in the United States, and about 477,300 cases of tumor were diagnosed in Germany every year, among which 125,790 cases chose TIVADs for chemotherapy [18]. At present, the clinical application of TIVAD is increasingly extensive, which is obviously better than other long-term intravenous infusion pathways and is the best choice for tumor patients, however, there are still many problems to be solved [30, 31].

## Conclusions

US-guided TIVADs via the right INV approach and the right IJV approach are both safe and reliable. There is a clear need for prospective and well-organized studies to confirm the feasibility and safety of the ultrasound-guided INV approach (both right and left) for TIVADs. It may stimulate future research in this area greatly.

## Data Availability

The datasets during the current study are available from the corresponding author on reasonable request.

## Abbreviations

CCA: Common carotid artery
CVC: Central vena catheterization
IJV: Internal jugular vein
INV: Innominate Vein
POS: Pinch-off syndrome
SCA: Subclavian artery
SCV: Subclavian vein
TIVAD: Totally implantable venous access device
